# Integrated Workflow for the Label-Free Isolation and Genomic Analysis of Single Circulating Tumor Cells in Pancreatic Cancer

**DOI:** 10.3390/ijms23147852

**Published:** 2022-07-16

**Authors:** Brittany Rupp, Sarah Owen, Harrison Ball, Kaylee Judith Smith, Valerie Gunchick, Evan T. Keller, Vaibhav Sahai, Sunitha Nagrath

**Affiliations:** 1 Department of Chemical Engineering, University of Michigan, Ann Arbor, MI 48109, USA; ruppb@umich.edu (B.R.); snowen@umich.edu (S.O.); hmball@umich.edu (H.B.); kjsm@umich.edu (K.J.S.); 2 BioInterface Institute, University of Michigan, Ann Arbor, MI 48109, USA; etkeller@med.umich.edu; 3 Division of Hematology and Oncology, Department of Internal Medicine, University of Michigan, Ann Arbor, MI 48109, USA; vagunchi@med.umich.edu (V.G.); vsahai@med.umich.edu (V.S.); 4 Rogel Cancer Center, University of Michigan, Ann Arbor, MI 48109, USA; 5 Department of Urology, University of Michigan, Ann Arbor, MI 48109, USA

**Keywords:** circulating tumor cells, pancreatic cancer, copy number variation, single-cell analysis

## Abstract

As pancreatic cancer is the third deadliest cancer in the U.S., the ability to study genetic alterations is necessary to provide further insight into potentially targetable regions for cancer treatment. Circulating tumor cells (CTCs) represent an especially aggressive subset of cancer cells, capable of causing metastasis and progressing the disease. Here, we present the Labyrinth–DEPArray pipeline for the isolation and analysis of single CTCs. Established cell lines, patient-derived CTC cell lines and freshly isolated CTCs were recovered and sequenced to reveal single-cell copy number variations (CNVs). The resulting CNV profiles of established cell lines showed concordance with previously reported data and highlight several gains and losses of cancer-related genes such as FGFR3 and GNAS. The novel sequencing of patient-derived CTC cell lines showed gains in chromosome 8q, 10q and 17q across both CTC cell lines. The pipeline was used to process and isolate single cells from a metastatic pancreatic cancer patient revealing a gain of chromosome 1q and a loss of chromosome 5q. Overall, the Labyrinth-DEPArray pipeline offers a validated workflow combining the benefits of antigen-free CTC isolation with single cell genomic analysis.

## 1. Introduction

Despite being the tenth most common cancer, pancreatic cancer is the third deadliest with an estimated 49,830 deaths a year [1,2]. Without many specific symptoms, over half of pancreatic cancer patients are diagnosed with the disease after it has become metastatic [1,3]. While the localized five-year survival rate is low (41.6%), the survival rate for metastatic patients is even lower (only 3%). This is due in part to limited treatment options, and patient and tumor heterogeneity [4]. Obtaining biopsies of the tumor can be an important step to understanding a patient’s cancer and providing personalized treatment [5]. Traditional tissue biopsies are invasive, difficult to obtain, and often are only performed at one time point. In the case of pancreatic cancer, the location of the pancreas within the body makes accessing the tumor and obtaining the biopsy or removing the cancer especially challenging [6]. Alternatively, liquid biopsies are less invasive and can be performed multiple times throughout the course of a patient’s treatment by any trained phlebotomist.

Liquid biopsies utilize biological markers found in the blood such as circulating tumor cells (CTCs). CTCs are cancer cells that are shed from a solid tumor into the bloodstream and circulate throughout the body. CTCs can provide real-time insight into tumor heterogeneity, metastatic potential, and patient-specific genetic variations [7,8]. With the advent of large-scale genomic sequencing, recent efforts have been made to accumulate and analyze whole cancer genomes across numerous cancer types [9,10]. Recent studies have found important trends in genomic instability and structural variation that indicated the evolution of cancer to malignancy [9,11,12]. Detection of genetic variations can be used to optimize cancer treatment and predict the risk of recurrence so that patients can receive better follow-up care [13,14].

Two common genetic variations researchers look at are single nucleotide variations (SNVs) and copy number variations (CNVs). CNVs research is promising, as CNVs often have a higher mutation rate than SNVs and affect a larger fragment of the genome resulting in more genomic changes that can be studied [15]. Additionally, since CNVs consist of 50 or more base pairs (bp) compared to SNV, which affect 1 bp, CNVs require fewer sequencing reads to determine significant results, decreasing the cost of the runs and allowing for more multiplexing of samples [16,17]. Using CTCs for CNV analysis would provide information at a single cell level that could be quickly isolated from patients. Previous work in pancreatic cancer CTC CNV analysis is currently very limited; however, the studies to date, such as that of Liu et al., use fluorescence in situ hybridization (FISH) to examine chromosome 8’s copy numbers exclusively [18]. Here, we report a method for isolating single CTCs from pancreatic cancer patients and preparing them for sequencing, which may allow for further investigation via the typically underutilized CNV analysis.

While CTCs are rare (10^0^–10^2^ CTCs compared to ~10^6^ white blood cells (WBCs) per milliliter of blood), technologies are available to isolate single CTCs for genomic analysis. The Labyrinth is a label-free, high-throughput microfluidic device that has been shown to isolate CTCs with a 90% success rate and deplete 90% of the WBCs in the sample while running at a flow rate of up to 2.5 mL/min [19]. As CTCs are isolated based on size and not by the phenotypic expression of surface proteins, the Labyrinth allows for a phenotypically diverse population of CTCs to be isolated from a large sample of blood. This is important for studying pancreatic cancer as the commonly used isolation marker, epithelial cell adhesion molecule (EpCAM), has varying expression levels in pancreatic cancer [20,21]. Khoja et al. further demonstrated this in pancreatic cancer CTCs by comparing a label-free and antibody-based isolation technology [21]. They found that the label-free method was not only able to detect CTCs in more patients but was also able to detect more CTCs in the samples.

Therefore, we developed a novel workflow that combines the Labyrinth CTC enrichment with the DEPArray Nxt system for isolation of single CTCs. The DEPArray Nxt System (Menarini Silicon Biosystems, Bologna, Emilia-Romagna, Italy) takes a cell solution and pushes it into a cartridge where cells are trapped in dielectrophoretic cages (DEP cages) [22]. Once in DEP cages, cells can be imaged and selected for isolation by creating a nonuniform electric field around the cell of interest. The machine then moves the field, thereby moving the cell. However, the DEPArray capacity is limited to the number of DEP cages available. Therefore, to investigate larger volumes of blood, samples must be sufficiently enriched for CTCs before being placed in the DEPArray. In this paper, we show for the first time that the Labyrinth can be used in conjunction with the DEPArray to enrich for CTCs and isolate single cells to perform low-pass sequencing for CNV detection and analysis.

## 2. Results

**Figure 1:**
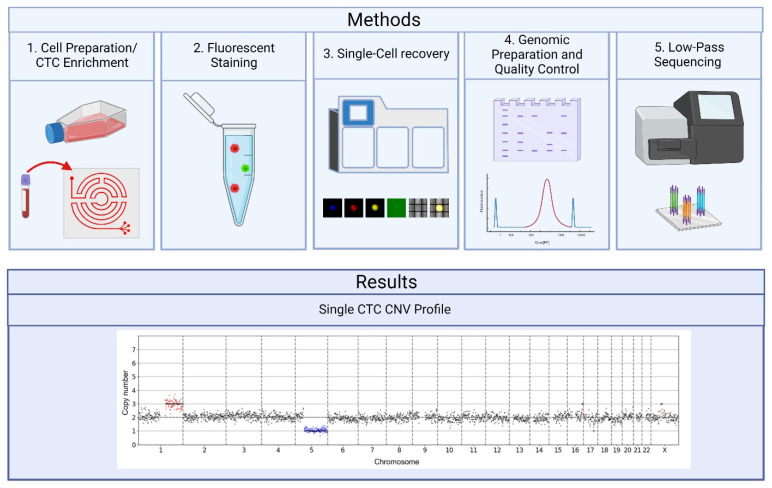
Study outline. 1: Cells are first obtained from culture (cell lines) or by processing blood through the Labyrinth to enrich for CTCs. 2: Cells are then fixed and fluorescently stained to allow for future cell identification. 3: The fixed cells are loaded onto the DEPArray for imaging and recovery of single cells. Cells are selected based on their fluorescent staining. 4: Single cells undergo whole genome amplification and library preparation to produce samples suitable for sequencing. The quality of the samples is confirmed after each step. 5: Samples are sequenced using an Illumina MiSeq. Results: Fastq files were submitted to MSBiosuite, which processed the data and provided single CNV profiles for each cell.

The proposed Labyrinth–DEPArray pipeline (Figure 1) was tested on established pancreatic cancer cell lines and pancreatic-cancer-patient-derived CTC cell lines. The pipeline was then validated using a blood sample obtained from a 53-year-old female with stage IV pancreatic ductal adenocarcinoma, who was undergoing chemotherapy. Cells were either obtained from culture or enriched using the Labyrinth before being stained and placed on the DEPArray for single-cell isolation. After single-cell isolation, cells underwent a whole genome amplification (WGA) and library preparation with appropriate QC to ensure sample quality before low-pass sequencing. The resulting Fastq files from sequencing were submitted to MSBiosuite (Menarini Silicon Biosystems, Bologna, Emilia-Romagna, Italy) for processing to obtain single-cell CNV profile data.

### 2.1. DEPArray Cell Selection and Recovery Based on Immunofluorescent Staining

**Figure 2:**
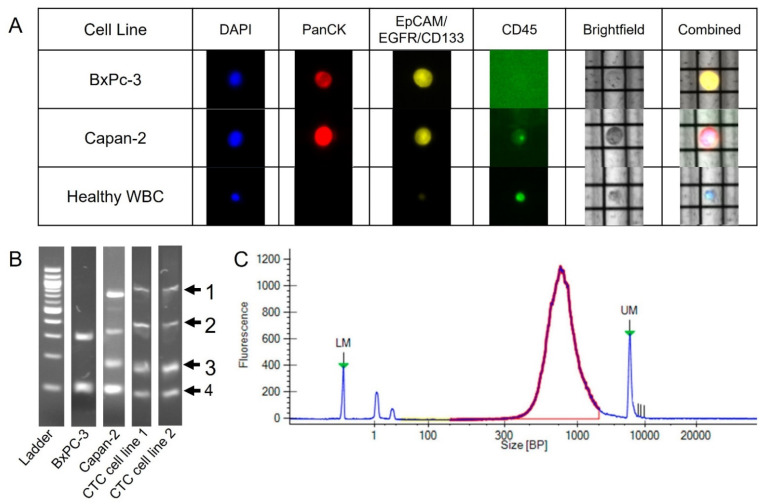
Examples of cell line cell results from the DEPArray, WGA QC, and library preparation QC. (**A**) Representative images of cells on the DEPArray. Cells were stained with PanCytokeratin, EpCAM/CD133/EGFR cocktail, CD45 and DAPI for identification and selection. (**B**) WGA QC results. Markers 1, 2, 3 and 4 indicate regions on the gel where bands should appear. Each region represents a different portion of the genome. (**C**) An example of post library preparation QC results. LM and UM correspond to lower and upper markers, respectively. The sample size is distributed in the expected size range of 100–2000 bp in purple/red. Blue lines indicate reading in the regions not expected to have sample.

Cells from three established pancreatic cancer cell lines, BxPC-3, Panc-1 and Capan-2, in addition to cells from two pancreatic cancer CTC-derived cell lines previously developed in the Nagrath lab, CTC cell line 1 and CTC cell line 2 were used as controls [23]. A staining protocol was developed to identify cells based on the presence or absence of pan-cytokeratin (PanCK), CD45 and other CTC surface markers: EpCAM, CD133 and epidermal growth factor receptor (EGFR). PanCK is a commonly used marker for positive identification of pancreatic CTCs while CD45 was used to identify WBCs [24]. However, the expression of PanCK on pancreatic CTCs has been shown to be heterogeneous [21]. Therefore, additional cancer surface markers were added to ensure that heterogeneous CTCs could be properly identified. The staining for additional CTC surface markers was performed using a cocktail of primary antibodies and one fluorescent secondary antibody. Cancer cell lines and CTCs were identified as PanCK and/or CTC surface marker+ and CD45−, and WBC were identified as PanCK/CTC surface marker- and CD45+. Each cell line was cultured, passaged and fluorescently labeled with the staining panel before being loaded into a DEPArray cartridge. After loading, the cartridge was inserted into the DEPArray where cells were distributed throughout the cartridge into individual dielectrophoretic cages using pressure. The entire chip was imaged in both fluorescence and bright field, using the DEPArray’s built-in camera, to identify cells. Figure 2A shows two cell lines cells and a healthy control WBC. Cells were identified based on the fluorescent images below and by the fluorescent intensities registered and recorded by the machine. Once cells of interest were identified, the cell was moved to the exit chamber for collection into a single tube for whole genome amplification. Two to three cells from each of the five cell lines were collected for downstream analysis.

### 2.2. Preparation for Sequencing Following the DEPArray

Following cell recovery, cells were whole-genome-amplified using the Ampli1 WGA kit (Menarini Silicon Biosystems, Bologna, Emilia-Romagna, Italy). QC was performed on all WGA cells using the Ampli1 QC kit (Menarini Silicon Biosystems, Bologna, Emilia-Romagna, Italy). The Ampli1 QC kit uses PCR to amplify, then test for the presence of four DNA fragments across different chromosomes, which is then used as an indicator of overall genomic quality. These fragments can be visualized as bands using gel electrophoresis (Figure 2B). The genome integrity index (GII) is defined as the number of bands visualized, with a GII of 4 indicating the highest quality. To perform low-pass sequencing for CNV analysis, the sample must have a minimum GII of 2. Of the cells selected for library preparation, four had a GII of 2, four a GII of 3 and six a GII of 4 (Table S1). In addition to Ampli1 QC, QC was also performed on all samples before and after library preparation. Cell line samples before library preparation had an average concentration of 26.24 ± 16.28 ng/μL (Table S1). After library preparation, libraries were quantified before sequencing as shown in Figure 2C. In this example, the red peak indicates the size and distribution of the fragments obtained from library preparation. The average size of the fragments generated across all cell line cells was 746 ± 233.79 bp (Table S1). These values are within the expected range of 100–2000 bp as reported by Menarini Silicon Biosystems.

### 2.3. Quality Control Considerations Using MSBiosuite

All samples that underwent library preparation were sequenced using an Illumina MiSeq, which outputted Fastq files for each sample. The resulting Fastq files were inputted into the MSBiosuite pipeline to obtain QC results and CNV data. There were three main metrics of QC used to evaluate each sample; a minimum of 200,000 mapped reads per sample, derivative log ratio spread (DLRS) and R50. DLRS is a calculation of the noisiness of the sample, where high values indicate high levels of noisiness and therefore potentially false CNVs. R50 is a measure of library complexity where low values indicate poor complexity and that certain regions may be favored over others during sequencing. Detailed descriptions of these QC metrics can be found in Section 4.11. Using these metrics, we filtered out low quality cells before further analysis. For the cell line samples, out of fourteen cells, two cells had DLRS values greater than 0.35, indicating a high noisiness, and were discarded. Two additional cells were also discarded due to too few mapped reads (Table S1).

### 2.4. CNV Data from Cell Lines

**Figure 3:**
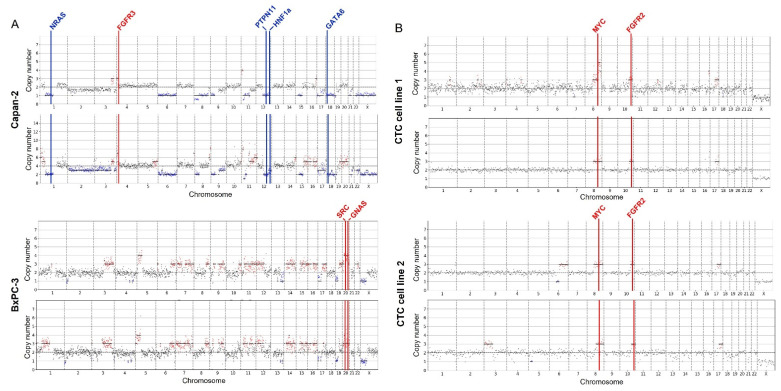
Single-cell copy number variation profiles from established pancreatic cancer cell lines and pancreatic-cancer-patient-derived CTC cell lines. Each graph represents an individual cell. Regions and genes highlighted in red experienced gains while regions and genes in blue experienced losses. Regions in black are neutral and center around the ploidy. Specific genes highlighted appeared across all individual cells (n = 2) of the same cell line and have been noted in reference datasets for established cell lines. Cell lines are as follows: (**A**) Capan-2, BxPC-3, (**B**) CTC cell line 1, CTC cell line 2.

As experimental controls, the five pancreatic cancer cell lines that were processed with the DEPArray were then amplified and sequenced. The CNV profiles from three established cell lines, BxPC-3, Capan-2 and Panc-1, were compared to published CNV data from CCLE and Nat. Bio [25,26]. Figure 3A shows the CNV profiles for the Capan-2 and BxPC-3 in duplicate cells (Panc-1, see Figure S2). The regions of gains and losses across the chromosomes are similar across cells despite differences in ploidy (Capan-2) and differences in the number of reads. The highlighted genes are cancer-specific genes where copy number variations were noted in both our single cells and the published cell line CNV data.

The remaining two cell lines are patient-derived CTC cell lines from Rivera-Baez et al. Our study is the first to do large scale genomic studies on these cell lines. Figure 3B shows the CNV profiles of the cell lines and highlights cancer-associated CNVs present in both cells of that cell line. Both cell lines show gains on parts of chromosome 8q (Myc), 10q (FGFR2) and 17q. Myc is a known oncogene that can both promote and repress other genes leading to cancer growth, and FGFR2 is associated with cell proliferation, migration and invasion of pancreatic ductal adenocarcinoma cells [27,28]. Copy number amplifications of Myc and FGFR2 have been reported in pancreatic cancer. Additional oncogenes/tumor suppressor genes in regions of gains and losses are identified in Table S2.

While the CNV profiles of the duplicate CTC cell line 1 cells appear to be in concordance, the CNV profiles of CTC cell line 2 appear to show minor variations between the two cells sequenced. One cell appears to have a 3p12.3–3p26.3 gain (CTNNB1), with the other showing a loss of 6q12–6q15 and gain of 6q16.1–6q27 (Myb, ESR1). As CTCs are heterogeneous, it is possible that cell lines derived from CTCs may show unique subpopulations. Further sequencing of more single cells is required to confirm the presence of unique subpopulations.

### 2.5. CNV Analysis of Pancreatic Patient Sample

**Figure 4:**
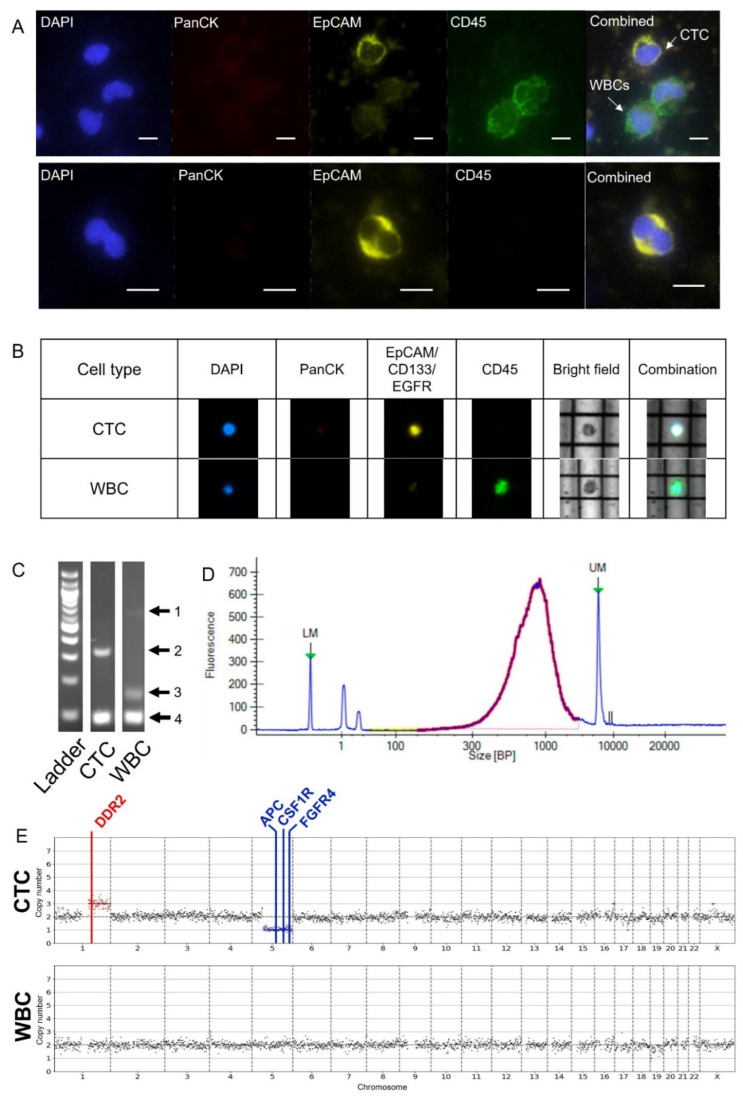
Staining, quality control, and CNV data for patient data. (**A**) Representative images of pancreatic patient CTCs and WBCs isolated from the Labyrinth and cytospun onto slides and stained. Cells were stained with DAPI, PanCytokeratin, EpCAM and CD45. Scale bar: 10 μm. (**B**) Representative images of pancreatic patient CTC and WBC on the DEPArray. Cells were stained with PanCK, EpCAM/CD133/EGFR cocktail, CD45 and DAPI for identification and selection. (**C**) WGA QC results. Markers 1, 2, 3 and 4 indicate regions on the gel where bands should appear. Each region represents a different portion of the genome. (**D**) An example of post library preparation QC results. LM and UM correspond to lower and upper markers, respectively. The sample size is distributed in the expected size range of 100–2000 bp in purple/red. Blue lines indicate reading in the regions not expected to have sample. (**E**) Copy number variation profiles of a CTC and a WBC isolated with the Labyrinth and DEPArray. Each graph represents an individual cell. Regions and genes highlighted in red experienced gains while regions and genes in blue experienced losses. Regions in black are neutral and center around a ploidy of 2. Genes from the MSBiosuite oncogenic panel with variations were highlighted.

As a proof of concept, we validated the Labyrinth–DEPArray protocol using blood from a metastatic pancreatic cancer patient. Enriched blood from the Labyrinth was divided into two portions: CTC enumeration and DEPArray processing. The sample for CTC enumeration was cytospun onto a microscope slide and stained using a similar staining panel as the DEPArray: PanCK, EpCAM, CD45 and DAPI. Figure 4A shows representative images of the CTCs identified. These CTCs appeared to be EpCAM+/PanCK-/CD45- with a concentration of 2.88 cells per milliliter of blood. The DEPArray sample was fixed and stained using the same staining panel as the DEPArray’ed cell lines. Figure 4B shows a CTC and WBC on the DEPArray. The CTC was identified based on strong staining of the CTC markers, EpCAM, CD133 and EGFR, and an absence of CD45.

After WGA, all patient sample cells with a GII of 2 or more underwent library preparation and sequencing. The average concentration of genomic material after WGA was 14.43 ng/μL and the average size of the fragments after library preparation was 507 bp (Table S1). Figure 4C,D show representative QC results for the patient sample. For sequencing, patient samples cells were sequenced on the same run as the cell lines and analyzed using the method previously described for the cell lines. All cells passed the three MSBiosuite QC metrics.

Using fluorescent staining, we identified one CTC with regions of gains and losses that were not seen in the matched WBC. (Figure 4E). An arm-level copy number gain can be noted in chromosome 1q as well as an arm-level copy number loss in chromosome 5q. A gain of 1q has been seen before in pancreatic cancer patients and arm-level CNVs have been identified in over a third of pancreatic ductal adenocarcinoma tumors [29]. DDR2 is located on chromosome 1q and has shown to be associated with cell proliferation and survival in multiple cancers including lung, melanoma and hepatoma [30]. Chromosome 5q is the location of oncogenic genes such as APC, CSF1R and FGFR4.

## 3. Discussion

The use of the Labyrinth to enrich for CTCs offers several benefits over other Menarini-approved preprocessing methods for the DEPArray. With a flow rate of up to 2.5 mL/min, the Labyrinth can process large volumes of blood using label-free isolation. In comparison, CellSearch, an FDA-approved technology, is designed to only process 7.5 mL of blood per run. Therefore, using the Labyrinth not only increases the number of CTCs that can be found but also allows for the opportunity to reduce the enrichment bias seen in other technologies that isolate cells based on surface markers. Cells collected using the Labyrinth–DEPArray pipeline are more representative of the heterogeneous population of CTCs and allow for a more thorough molecular profiling of each patient’s tumor. Our study shows that the proposed pipeline can be used to detect CNVs on a single CTC scale.

CNVs have been highly correlated to differential gene expression, and new databases are working to integrate patient demographic and CNV data to investigate possible cancer-related CNVs [31,32]. While some CNV detection and analysis has been performed in certain cancers including breast and colorectal, limited work has been done with respect to pancreatic cancer [18,33,34,35,36,37]. Pancreatic cancer patients’ CNVs have largely been limited to detection in primary patient tumor tissues, patient-derived cell lines and circulating tumor DNA (ctDNA), each accompanied by multiple limitations [9,35,36]. Primary patient tumor tissue may not always represent full tumor heterogeneity and is often not resectable in patients with metastatic cancer, making it difficult to obtain for analysis [38,39]. If enough material can be obtained to develop cell lines, they take considerable time to develop, and can develop mutational changes during development that do not reflect the original cells [40]. While ctDNA is obtained via a liquid biopsy, making collection easier, it can be quickly degraded in circulation leading to highly fragmented pieces that cannot be used to detect larger CNVs [41]. ctDNA also suffers from low concentrations with surrounding cell-free DNA from healthy cells further introducing conflicting signals and complicating analysis. This study aimed to overcome these limitations using single CTCs, which can provide readily accessible genomic information on a given patient’s cancer.

Previous work comparing WGA methods on single cell line cells found an average concentration of 19.2 ± 6.9 ng/μL per sample after Ampli1 WGA [42]. Utilizing the Labyrinth–DEPArray pipeline, we reported an average concentration of 26.24 ± 16.28 ng/μL for cell line cells and 14.43 ng/μL for patient sample cells using the same WGA method. These results were promising as they showed cells recovered using our process had similar concentrations to previously reported results. The average size of fragments generated using our process was 746 ± 232.79 bp for cell line cells and 507 bp for patient sample cells, which is within the expected range of 100–2000 bp. Both the average concentration and size was lower in patient sample cells compared to cell lines cells. This was expected as patient sample cells are less resilient to changing conditions than cell lines and had to undergo additional processing which could lead to cell death and DNA degradation. However, the concentration and sizes were still within an acceptable range and sequencing of patient sample cells yielded good quality results indicating that our pipeline was capable of overcoming challenges associated with CTCs isolated from patient samples.

To detect CNVs, low-pass sequencing was used instead of traditional microarrays. Previously, microarrays were commonly used for CNV detection as they allowed for screening of specific regions and were much cheaper than sequencing. Both referenced cell line data sets used for comparison were obtained using arrays. The CCLE data set and the Klijn et al. data set were published in 2012 and 2015, respectively [25,26]. However, array-based technologies have presented limitations such as varying probe density across different platforms and differences in detection approaches between technologies (array-based comparative genomic hybridization (aCGH) and single-nucleotide polymorphism arrays (SNParrays)) [43,44]. With the improvements in sequencing technology and the resulting decrease in costs, low-pass sequencing has now become a viable option for CNV detection. The methods described in this paper not only demonstrate the necessary steps for sequencing but a compatible and easy method for data analysis.

The resulting sequenced files were processed using MSBiosuite. Cells removed from analysis after the sequencing step failed one of two possible MSBiosuite QC metrics: high (poor) DLRS or lower than 200,000 reads per cell. For samples with low DLRS numbers, the most likely cause was degradation of the samples over time. The cells with low DLRS numbers came from DEPArray runs that were performed several months before Ampli1 and sequencing could be performed. For the patient sample, the sample was able to be processed and sequenced within the span of 2 months and had no cells with high DLRS numbers. This supports the idea that samples should be processed as soon as possible to reduce degradation and yield the best chance of recovering data. For the cell lines with lower than 200,000 reads, both cells were from the same DEPArray run. Cells from this run saw a low percentage of reads mapped to the human genome suggesting a contamination of the sample, which would explain the low number of mapped reads despite over 500,000 reads being sequenced for the sample.

To further understand the significance of the variations found in the CTC-derived cell lines and the patient’s CTC, we used CBioPortal to compare the discovered CNVs to published CNVs in pancreatic adenocarcinoma tissue samples. Using CbioPortal, we examined CNV data from 1011 previously sequenced pancreatic adenocarcinoma tissue samples with a subset of 269 metastatic pancreatic adenocarcinoma samples from nine studies [9,45,46,47,48,49,50,51,52,53]. This allowed us to examine similarities between tissue samples and CTC samples. Myc was found in both data sets with a frequency rate of 4.4% in all tissue samples and 4.8% in metastatic samples only. Myc was the third most common amplification in the metastatic dataset, which supports previous research showing the association between Myc amplification and poor patient outcomes [54]. While the amplification of Myc was not seen in patient sample CTC, it was seen in both CTC-derived cell line cells, CTC cell line 1 and CTC cell line 2, and supports continuing research focused on targeting Myc [38,55].

In the patient sample CTC, two arm-wide copy number alterations were observed: a gain of chromosome 1q and a loss of chromosome 5q. This agrees with previously published data estimating 25% of a typical tumor tissue is affected by arm-level alterations; however, exact conclusions are difficult to make by examining arm-level alterations alone. [56]. By further examining genes located on these chromosomes, we were able to observe a copy number loss of APC. APC is a tumor suppressor gene that is known to affect Wnt/β-catenin signaling and CD34 expression [57,58]. APC loss leads to an increase in CD34 expression which is linked to increases in cell invasion and migration. Additionally, it has been shown that APC haploinsufficiency coupled with P53 deletion and KRASG12D activation can lead to the development of pancreatic intraepithelial neoplasia lesions in mice models with further progression to pancreatic ductal adenocarcinoma, demonstrating the importance of understanding variations in APC [57].

While larger scale studies are needed to validate the findings in the CTC cell lines and patient samples, this work demonstrates a capable workflow for obtaining genomic information from single CTCs. As a proof of concept, this work only looked at CNVs using low-pass sequencing. However, future work could use this single CTC isolation method to examine other genomic alterations of interest to researchers. The current workflow is limited by the sample processing throughput. While the Labyrinth allows for the efficient processing of large blood volumes, a lack of multiplexing in both the Labyrinth and the DEPArray inhibits the ability to process multiple samples at once, limiting the diagnostic capabilities of this workflow in a clinical setting. Nevertheless, additional genomic analysis of diverse metastatic pancreatic cancer CTCs may provide insight into targetable pathways to improve patient treatment and survival. Applying this workflow to larger patient cohorts may yield additional information about inter- and intra-patient heterogeneity and reveal CNVs important to metastasis.

## 4. Materials and Methods

### 4.1. Cell-Line/Patient-Derived CTC Line Culture and CellTracker Staining

All cell lines were cultured at 37 °C, 5% CO_2_ under normoxic conditions. The Panc-1, Capan-2 and BxPC-3 cell lines were obtained from ATCC. The pancreatic cancer CTC cell lines, CTC cell line 1 and CTC cell line 2, were developed in-house and previously published by Rivera-Báez et al. [23]. Panc-1 and Capan-2 cells were maintained in Dulbecco’s Modified Eagle’s Medium (DMEM) (Gibco, Waltham, MA, USA) supplemented with 10% fetal bovine serum (FBS) (Sigma, St. Louis, MO, USA), and 1% antibiotic-antimycotic (anti-anti) (Gibco, Waltham, MA, USA). CTC cell line 1, CTC cell line 2 and BxPC-3 were maintained in RMPI-1640 (Gibco, Waltham, MA, USA) supplemented with 10% FBS and 1% anti-anti. Cells were grown to 70–80% confluence before subculturing using 0.05% Trypsin-EDTA (Gibco, Waltham, MA, USA). Between cell passages, media were replaced every 48–72 h. All cell lines were tested and reported negative for mycoplasma using MycoAlert^TM^ Mycoplasma Detection Kit (Lonza, Basel, Switzerland).

For experiments using prelabeled cells, cells were dyed using CellTracker^TM^ Green CMFDA dye (Invitrogen, Waltham, MA, USA), and CellMask^TM^ orange dye (Invitrogen, Waltham, MA, USA). CellTracker^TM^ dyes were used at 2 μM in serum-free media for 30 min, followed by a 30 min incubation in complete media. CellMask^TM^ was used at a 1× concentration in serum free media for 10 min. All samples were washed 3× to remove excess dye before fixation with 2% paraformaldehyde (PFA) for 20 min.

### 4.2. Patient Enrollment

The patient was a 53-year-old Caucasian non-Hispanic female diagnosed with stage IV pancreatic ductal adenocarcinoma and undergoing chemotherapy. The patient gave informed consent to participate in the research study. They were enrolled in the Assessment of Biological Markers in Pancreatic Cancer study (HUM00025339). This study protocol was approved by the University of Michigan Medicine Institutional Review Board.

### 4.3. Labyrinth Fabrication

Labyrinths were manufactured by mixing Sylgard 184 silicone elastomer base and curing agent (Ellsworth Adhesive, Germantown, WI, USA) at a ratio of 10:1 and pouring the liquid polymer into a Labyrinth master mold, fabricated using standard photolithography methods with SU8. Molds were placed under vacuum for 1 h to remove residual bubbles and then baked in an oven at 65 °C to solidify the polydimethylsiloxane (PDMS). Once solidified, the PDMS was cut from the master mold, 0.75 um holes were punched at the inlets and outlets of the device, and the PDMS was plasma-bonded to a 75 × 50 mm glass slide. Labyrinths were placed on a hot plate at 85 °C for 15 min, allowed to cool, and 10-inch tubing was placed in the inlets and outlets of the device.

### 4.4. Sample Collection and Processing

Blood was collected into EDTA tubes and processed within 2 h of sample collection. For CTC isolation prior to DEPArray collection, the blood was processed using Ficoll-PaqueTM PLUS (GE Healthcare, Chicago IL, USA) to enhance RBC depletion following the manufacturer’s protocol. After centrifugation, the plasma and nucleated cell layers were collected and diluted to 5× the original blood volume with phosphate buffered saline pH 7.4 (PBS) (Gibco, Waltham, MA, USA). The sample was processed through the Labyrinth at 2000 µL/min. Prior to sample collection, flow was stabilized for 1 mL of processing, then the CTC outlet (Outlet 2) was collected in a separate conical. The resulting CTC-enriched sample was processed a second time in the Labyrinth, following the same procedure. Then, 250 μL of the second Labyrinth product was taken for CTC enumeration and the remaining portion was centrifuged at 400× *g* for 5 min, and the supernatant was removed. The sample was fixed in 2% paraformaldehyde (PFA) (Thermo Fisher Scientific, Waltham, MA, USA) for 20 min. The sample was centrifuged using the same conditions to wash the PFA from the sample. The sample was stored in PBS at 4 °C until suspension staining could be performed.

### 4.5. Immunofluorescent Staining and CTC Enumeration

After Labyrinth processing was completed, 250 μL of the second outlet product was cytospun onto polysine-coated glass slides (Fisher Scientific, Waltham, MA, USA). Slides were fixed with 4% PFA and stored at 4 °C until staining could be performed. Slides were permeabilized with 0.2% Triton X-100 (Sigma-Aldrich, St. Louis, MO, USA) for 3 min. After removing the triton and washing the slides, the slides were blocked with 200 μL of 10% goat serum (Thermo Fisher Scientific, Waltham, MA, USA) for 30 min. After blocking, primary antibodies were added and allowed to incubate at 4 °C overnight: 1:100 dilution of anti-CD45 (BioRad, Hercules, CA, USA), 1:200 dilution of anti-pan cytokeratin (BioRad, Hercules, CA, USA), 1:100 dilution of anti-vimentin (Cell signaling, Danvers, MA, USA) and 1:10 dilution of anti-EpCAM (R&D system, Minneapolis, MN, USA) in 10% goat serum. The following day, primaries were washed off the slides and secondary antibodies were added and incubated for 45 min at room temperature: 1:500 dilution of AF488 (Invitrogen, Waltham, MA, USA), AF546 (Invitrogen), AF647 (Invitrogen) and a 1:10 dilution of AF750 (Invitrogen) in 10% goat serum. Secondary antibodies were then washed from the slides and ProLong gold antifade mountant with DAPI (Invitrogen, Waltham, MA, USA) and a coverslip were added. Slides were fluorescently imaged on a Nikon Ti eclipse and EpCAM+ and/or PanCK+/DAPI+/CD45- cells were counted as CTCs.

### 4.6. Suspension Staining

Previously fixed samples were centrifuged at 400× *g* for 10 min to remove PBS. Cells were resuspended in 100 μL of 1% bovine serum albumin (BSA) (Sigma, St. Louis, MO, USA) and incubated for 10 min. Samples were centrifuged again to remove BSA before being resuspended in 90 μL of Running Buffer (Miltenyi Biotec, Bergisch Gladbach, Germany). A primary antibody staining cocktail of the following biotinylated antibodies was added and incubated for 15 min at 4 °C: 10 μL of anti-EpCAM (R&D system, Minneapolis, MN, USA), 10 μL of anti-CD133 (Miltenyi Biotec, Bergisch Gladbach, Germany), 5 μL of anti-EGFR (RayBiotech, Peachtree Corners, GA, USA) and 20 μL anti-CD45 (BioRad, Hercules, CA, USA). After 15 min, the reaction was quenched with 1 mL of running buffer before being centrifuged. The supernatant was discarded and the following solutions were added before incubating an additional 10 min at 4 °C: 90 μL of Inside Stain (Miltenyi Biotec, Bergisch Gladbach, Germany), 2.5 μL of strepavidin Alexa Fluor 488 conjugated (Invitrogen, Waltham, MA, USA), 10 μL of goat anti-mouse IgG2a AF647 (Invitrogen, Waltham, MA, USA), 10 μL of PE anti-pan cytokeratin (Abcam, Cambridge, UK) and 2.5 μL of DAPI (Invitrogen, Waltham, MA, USA). After incubation, the reaction was quenched with 1 mL of running buffer and centrifuged.

### 4.7. DEPArray

The samples were centrifuged at 400× *g* for 10 min before removing the supernatant and replacing it with SB115 (Menarini Silicon Biosystems, Bologna, Emilia-Romagna, Italy), which ensures the right conductivity for cell routing and collection. This was repeated to ensure complete replacement of the buffer. Additional SB115 was pipetted into 1.5 mL Eppendorf tubes and sealed with parafilm before being placed in the sonicator for a minimum of 15 min to remove all air bubbles.

After preparation, the SB115 and sample were loaded onto the DEPArray™ following the manufacturer’s instruction for cartridge loading. The machine then distributed the cells throughout the cartridges and imaged the sample based on user-defined channels and focal plane. Here, the chip was scanned in DAPI, FITC, PE, and APC fluorescent channels, and bright field. The user then selected cells to be isolated from the machine. The DEPArray manipulated the electric field around individual cells to route them off of the machine into 0.2 mL qPCR tubes. Once collected, the liquid volume was reduced to approximately 2 μL using the VRNxt (Menarini Silicon Biosystems, Bologna, Emilia-Romagna, Italy) and the sample was stored for further analysis.

### 4.8. Whole Genome Amplification (WGA) and Associated Quality Control (QC)

Whole genome amplification (WGA) was performed using the Menarini Silicon Biosystems’ Ampli1 whole genome amplification kit following the manufacturer’s protocol. Briefly, the cells were lysed, then the DNA was digested, annealed and ligated before PCR was performed. Following PCR, the samples were QC’ed and prepared for low-pass sequencing by library preparation.

To perform the QC, 1 μL of the 50 μL WGA final product and control gDNA was taken and QC was performed using the Menarini Silicon Biosystems’ Ampli1 QC kit following the manufacturer’s protocol. As part of the QC, PCR was done to amplify four different genomic targets which were then seen on a gel to confirm WGA was performed correctly. A 3% agarose gel was made by combining 25 mL of 1× Tris-acetate-EDTA (TAE) buffer (Lonza, Basel, Switzerland), 2.5 μL of SYBR Safe DNA gel stain (Invitrogen, Waltham, MA, USA), and 0.75 g of UltraPure agarose powder (Invitrogen, Waltham, MA, USA) in a beaker, swirling and letting the solution sit for 5 min. The solution was heated in a microwave in 30 s intervals and swirled between run times until the agarose powder was completely dissolved, approximately 1 min. After allowing the solution to cool for 2 min, the gel was poured into a mold and set for 45 min before being placed in a gel box and covered with TAE buffer.

To load the gel, 10 µL of sample and 2 µL of Gel Loading Dye, Purple (6×) no SDS (New England Biolabs, Ipswich, MA, USA) were mixed and 5 μL of the sample solution was pipetted into each lane. For the ladder, 2 µL of Tris-EDTA (TE) buffer (Corning, Corning, NY, USA), 1 µL of gel loading dye and 1 µL of DNA ladder (New England Biolabs, Ipswich, MA, USA) were combined, then 3 µL of ladder solution was pipetted into the lane. Gels were run at 120 V and 3 A for 30 min and imaged using the FluroChem M imaging system (Protein Simple, Santa Clara, CA, USA). The genome integrity index (GII) is defined as the number of bands visualized on the QC gel per sample. The GII can range from 0 to 4, with 4 indicating the highest quality. Samples with a GII of 2 or more were prepared for sequencing.

### 4.9. Low-Pass Preparation and Illumina Sequencing

Library preparation and sequencing were performed at the University of Michigan Advanced Genomics Core. The Ampli1 LowPass kit for Illumina (Menarini Silicon Biosystems, Bologna, Emilia-Romagna, Italy) was used to barcode and prepare each sample for sequencing. The sample preparation was done according to the manufacturer’s protocol. First, 5 μL of Ampli1 WGA product was first cleaned up using SPRIselect beads (Beckman Coulter, Brea, CA, USA). Next, two barcoding mixes were prepared and added to each individual sample in series to uniquely label each sample so it could be later pooled. After barcoding, the library was amplified and cleaned up. Libraries were quantified using the Perkin-Elmer LabChip (PerkinElmer, Waltham, MA, USA).

All samples were run on the Illumina MiSeq platform using the MiSeq Reagent Kit v3 (Illumina, San Diego, CA, USA). The low-pass libraries were denatured and diluted according to the manufacturer’s protocol. Due to the library preparation with the low-pass kit, 5 μL of the Ampli1 SEQ primer must be added to 595 μL of HT1 Illumina buffer and loaded on the reagent cartridge. The samples were run for 150 cycles of single-read sequencing.

### 4.10. Single-Cell Sequencing Data Analysis

Raw data from the Illumina sequencer, Fastq files, were processed with MSBiosuites, a cloud-based platform accessible using a web-based GUI, courtesy of Menarini Silicon Biosystems. Files were uploaded to MSBiosuites with corresponding metadata including patient sex, number of cells (n = 1 for single cell inputs) and GII. Once the information was uploaded, samples were submitted for analysis. Each sample was mapped to the human reference genome hg19 and reads from alternative chromosomes and low-quality regions were removed. Only samples with greater than 200,000 mapped reads were processed. After processing, sample QC and CNV data for all 23 chromosomes and gene-level copy number status were outputted for all samples.

### 4.11. MSBiosuite Specific QC Metric

Derivative log ratio spread (DLRS) is a measure of noisiness of the data. DLRS is calculated on a random subset of 200,000 mapped reads. The copy number of these reads is then calculated using a window size of 1 mega base pair (Mbp) and ploidy of 2. This subset of copy numbers is then used to calculate DLRS using the following formula, where diff is the nth discrete difference between consecutive windows along the genome: DLRS = standard deviation ((diff(*log*_2_(Copy Number/2)))/√2).

R50 is a measure of library complexity and is calculated by sorting the number of Ampli1 fragments, sorted by most covered and dividing the number of fragments covered by 100,000 reads by the number covered by 200,000 multiplied by 100.

## 5. Conclusions

The Labyrinth–DEPArray pipeline offers the ability to isolate single CTCs for downstream analysis. Cells isolated using this pipeline show sufficient quality at each step of the CNV downstream processing. We were able to observe similar CNV profiles across multiple established cell lines and note new CNVs in patient-derived CTC cell lines and fresh CTCs. As CTCs are part of the metastatic cascade, developing a deeper understanding of them and their interaction with other mutations will lead to a more thorough understanding of pancreatic cancer and possibly the development of further therapeutic agents.

## Data Availability

The data presented in this study are openly available in the BioProject database, BioProjectID [PRJNA858814].

## Supplementary Materials

The following supporting information can be downloaded at: https://www.mdpi.com/article/10.3390/ijms23147852/s1.
