# Supplementary material for: Integrated Workflow for the Label-Free Isolation and Genomic Analysis of Single Circulating Tumor Cells in Pancreatic Cancer
Source: Int J Mol Sci. 2022 Jul 16;23(14):7852. doi: 10.3390/ijms23147852 (PMC9316651; doi:10.3390/ijms23147852)
Supplement: Supplementary file 1 [file ijms-23-07852-s001.zip › ijms-1815683-supplementary.pdf]

International Journal of
Molecular Sciences

MDPI

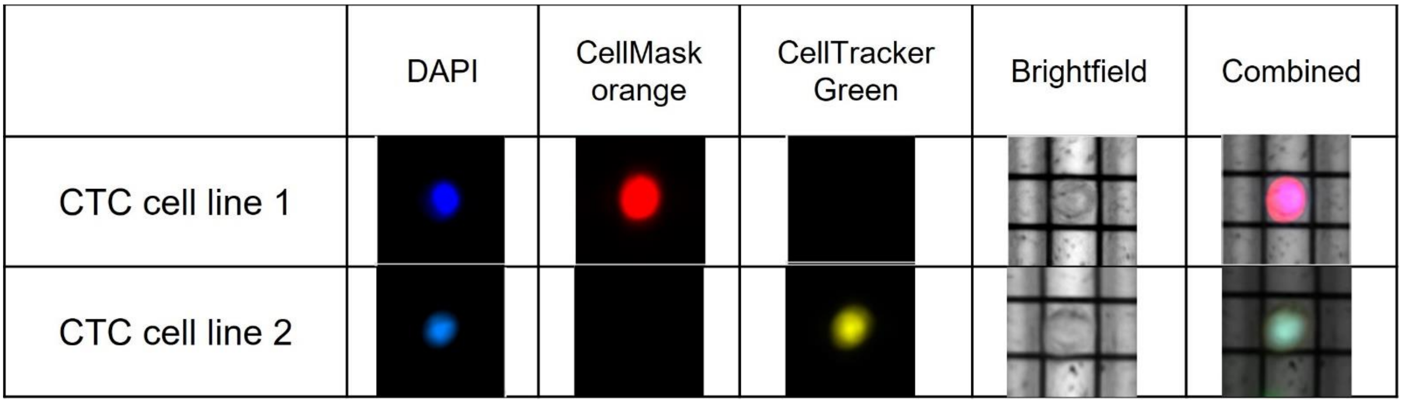

Supplementary Figure S1: Staining of the pancreatic patient derived CTC cell lines on the DEPArray. Each cell line was fluorescently stained and loaded into a DEPArray cartridge. The unique fluorescent dye allowed for each cell type to be identified and multiple cell lines to be pooled and multiplexed, reducing time and necessary reagents.

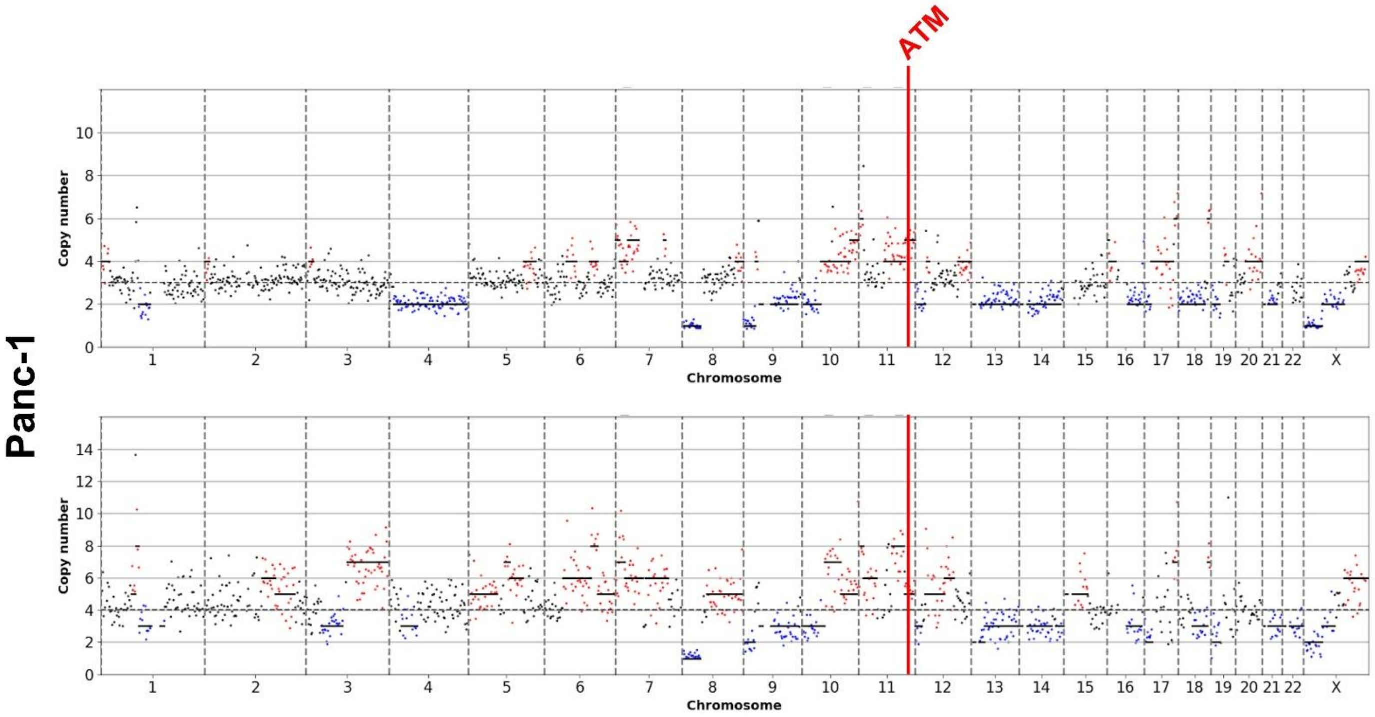

Supplementary Figure S2: Single cell copy number variation profiles of Panc-1 cells. Each graph represents an individual cell. Regions and genes highlighted in red experienced gains while regions and genes in blue experienced losses. Regions in black are neutral and center around the ploidy. Specific genes highlighted appeared across all individual cells (n=2) and have been noted in reference datasets.

Supplementary Table S1. Patient characteristics

Age	Gender	Race	Ethnicity	Cancer	Stage	Treatment
53	Female	Caucasian	Non-Hispanic	Pancreatic Ductal Adenocarcinoma	IV	Chemotherapy (Folfirinox)

Supplementary Table S2. Quality control values for cells submitted to sequencing. Number of WGA bands and concentration were determined after whole genome amplification, average fragment size was determined after library preparation and the number of mapped reads, DLRS and R50 were determined after sequencing. Values highlighted in red were below thresholds and the corre-sponding cells were discarded from further analysis.

Sample	# of WGA bands	Concentration (ng/µl)	Average fragment size (bp)	Mapped reads	DLRS	R50
BxPC3	2	14.3	517	462,300	0.276	44
	2	10.5	483	655,500	0.383	31
	2	21.8	492	726,400	0.278	44
Capan-2	4	10.7	508	397,900	0.235	45
	4	12.4	538	584,200	0.245	44
Panc-1	4	8.48	786	451,200	0.321	41
	3	26.2	729	382,200	0.282	42
	3	41.6	786	505,400	0.554	43
CTC cell line 1	3	49.8	1035	204,100	0.237	46
	4	41.2	1059	<200,000	--	
	2	5.44	575	529,600	0.294	38
CTC cell line 2	3	41.6	1080	203,300	0.228	46
	4	50.8	1043	202,500	0.278	47
	4	31.6	813	<200,000	--	--
CTC	2	19.6	482	651,400	0.217	47
WBC	2	9.26	532	609,600	0.23	45

Supplementary Table S3: Cancer related genes identified by in regions of gains or loss of the patient derived CTC cell lines. Cancer related genes were identified by MSBiosuite. For each gene the following information is given: chromosome location, identification as either an oncogene or tumor suppressor gene, CNV gain or loss, number of CTC cell line 1 cells the variation was found in and number of CTC cell line 2 cells the variation was found in (max n=2 for each cell line).

Chromosome	Chromosome Location	Gene	Oncogenes/ Tumor Suppressor	Gain/ Loss	CTC cell line 1	CTC cell line 2
1	1p36.32	TNFRSF14	Tumor Suppressor	Gain	1	--
	1p36.31	RPL22	Oncogene	Gain	--	1
	1p36.22	PIK3CD	Oncogene	Gain	--	1
	1p36.22	MTOR	Oncogene	Gain	--	1
	1p36.12	EIF4G3	Oncogene	Gain	--	1
	1p36.13	SDHB	Tumor Suppressor	Gain	1	1
	1q31.2	B3GALT2	Tumor Suppressor	Gain	1	--
	1q31.3	ASPM	Oncogene	Gain	1	--
	1q31.3-q32.1	PTPRC	Oncogene	Gain	1	--
	1q43	OPN3	Tumor Suppressor	Gain	--	1
2	2q32.2	ORMDL1	Tumor Suppressor	Gain	1	--
	2q36.1	ACSL3	Oncogene	Gain	1	--
	2q36.2	CUL3	Tumor Suppressor	Gain	1	--
3	3p26.2	CRBN	Tumor Suppressor	Gain	--	1
	3p26.2	CRBN	Tumor Suppressor	Loss	1	--
	3p25.3	FANCD2	Tumor Suppressor	Gain	--	1
	3p25.3	VHL	Tumor Suppressor	Gain	--	1

	3p25.3	SRGAP3	Oncogene	Gain	--	1
	3p25.2	PPARG	Oncogene	Gain	--	1
	3p25.2	MKRN2	Oncogene	Gain	--	1
	3p25.1	XPC	Tumor Suppressor	Gain	--	1
	3p24.1	TGFBR2	Tumor Suppressor	Gain	--	1
	3p21.31	RHOA	Oncogene	Gain	--	1
	3p21.31	SETD2	Tumor Suppressor	Gain	--	1
	3p21.31	RBM5	Tumor Suppressor	Gain	--	1
	3p22.3	CLASP2	Tumor Suppressor	Gain	--	1
	3p22.2	MLH1	Tumor Suppressor	Gain	--	1
	3p22.2	ITGA9	Oncogene	Gain	--	1
	3p22.2	MYD88	Oncogene	Gain	--	1
	3p22.1	CTNNB1	Oncogene	Gain	--	1
	3p21.1	BAP1	Tumor Suppressor	Gain	--	1
	3p21.1	PBRM1	Tumor Suppressor	Gain	--	1
	3p14.3	WNT5A	Oncogene	Gain	--	1
	3p13	MITF	Oncogene	Gain	--	1
	3p13	FOXP1	Oncogene	Gain	--	1
4	4q31.21	INPP4B	Tumor Suppressor	Gain	1	--
5	5p15.33	SDHA	Tumor Suppressor	Gain	1	--
	5p15.33	TERT	Oncogene	Gain	1	--
6	6q13	EEF1A1	Tumor Suppressor	Loss	--	1
	6q14.3	ZNF292	Tumor Suppressor	Loss	--	1
	6q21	PRDM1	Tumor Suppressor	Gain	--	1
	6q21	HDAC2	Tumor Suppressor	Gain	--	1
	6p21.1	TFEB	Oncogene	Gain	1	--
	6p21.1	CCND3	Oncogene	Gain	1	--
	6p21.1	TRERF1	Oncogene	Gain	1	--
	6q22.1	ROS1	Oncogene	Gain	--	1
	6q23.2	ARG1	Tumor Suppressor	Gain	--	1
	6q23.3	TNFAIP3	Tumor Suppressor	Gain	--	1
	6q23.3	MYB	Oncogene	Gain	--	1
	6q23.3	BCLAF1	Oncogene	Gain	--	1
	6q24.1	ECT2L	Tumor Suppressor	Gain	--	1
	6q25.1-q25.2	ESR1	Oncogene	Gain	--	1
	6q25.3	ARID1B	Tumor Suppressor	Gain	--	1
	6q25.3	IGF2R	Tumor Suppressor	Gain	--	1
	6q26	MAP3k4	Oncogene	Gain	--	1
7	7p22.3-p22.2	GNA12	Tumor Suppressor	Gain	1	--
	7p11.2	EGFR	Oncogene	Loss	1	--
8	8q13.3	NCOA2	Oncogene	Gain	2	1
	8q21.3	NBN	Tumor Suppressor	Gain	2	2
	8q22.2	COX6C	Oncogene	Gain	2	2
	8q24.11	MIR3610	Tumor Suppressor	Gain	2	2
	8q24.11	RAD21	Tumor Suppressor	Gain	2	2
	8q24.11	EXT1	Tumor Suppressor	Gain	2	2

	8q24.21	MYC	Oncogene	Gain	2	2
	8q24.22	NDRG1	Oncogene	Gain	2	2
9	9q34.2	BRD3	Oncogene	Gain	1	--
10	10q24.32	SUFU	Tumor Suppressor	Gain	2	2
	10q25.2	TCF7L2	Oncogene	Gain	2	2
	10q25.2	SNORA87	Oncogene	Gain	2	2
	10q26.13	FGFR2	Oncogene	Gain	2	2
	10q26.3	MGMT	Oncogene	Gain	2	2
11	11p13	CAT	Oncogene	Gain	1	--
12	12q21.32	C12orf29	Tumor Suppressor	Gain	1	--
13	13q34	IRS2	Tumor Suppressor	Gain	1	--
	13q34	ING1	Tumor Suppressor	Gain	1	--
17	17q21.33	SPOP	Oncogene	Gain	2	2
	17q21.33	COL1A1	Oncogene	Gain	2	2
	17q22	HLF	Oncogene	Gain	2	2
	17q22	RNF43	Tumor Suppressor	Gain	2	2
	17q22	RAD51C	Tumor Suppressor	Gain	2	2
	17q22	MIR142	Tumor Suppressor	Gain	2	2
	17q23.1	CLTC	Oncogene	Gain	1	2
	17q23.2	BRIP1	Tumor Suppressor	Gain	1	2
	17q23.3	PPM1D	Tumor Suppressor	Gain	1	2
	17q23.3	CD79B	Oncogene	Gain	2	2
	17q23.3	DDX5	Oncogene	Gain	2	2
	17q23.3	CEP95	Oncogene	Gain	2	2
	17q24.1	GNA13	Tumor Suppressor	Gain	1	2
	17q24.1	AXIN2	Tumor Suppressor	Gain	2	2
	17q24.2	ARSG	Tumor Suppressor	Gain	2	2
	17q24.2	BPTF	Oncogene	Gain	2	2
	17q24.3	SOX9	Tumor Suppressor	Gain	2	2
	17q25.1	MFSD11	Oncogene	Gain	2	2
	17q25.2	SRSF2	Oncogene	Gain	2	2
	17q25.3	SEPT9	Oncogene	Gain	2	2
	17q25.3	CANT1	Oncogene	Gain	2	2
	17q25.3	ENDOV	Oncogene	Gain	2	2
	17q25.3	ACTG1	Oncogene	Gain	2	2
	17q25.3	ASPSCR1	Oncogene	Gain	2	2
	17q25.3	RNF213	Oncogene	Gain	--	2
	17q25.3	B3GNTL1	Tumor Suppressor	Gain	1	--
20	20q13.32	GNAS	Oncogene	Gain	1	--
	20q13.33	SS18L1	Oncogene	Gain	1	--
